# Men’s knowledge and attitudes about cervical cancer screening in Kenya

**DOI:** 10.1186/s12905-014-0138-1

**Published:** 2014-11-22

**Authors:** Joelle I Rosser, Jennifer M Zakaras, Sabina Hamisi, Megan J Huchko

**Affiliations:** Department of Internal Medicine, University of Washington, 1959 NE Pacific Street, Box 356421, Seattle, WA 98195-6421 USA; Department of Obstetrics, Gynecology, and Reproductive Sciences, University of California, San Francisco, San Francisco General Hospital, 1001 Potrero Avenue, Ward 6D-14, San Francisco, CA 94110 USA; Family AIDS Care and Education Services, Kenya Medical Research Institute, PO BOX 54840-00200, Mbagathi Way, Nairobi City, Kenya

**Keywords:** Cervical cancer prevention, Health knowledge and attitudes, Men, HIV, Women’s global health, Sub-Saharan Africa

## Abstract

**Background:**

A number of studies have identified male involvement as an important factor affecting reproductive health outcomes, particularly in the areas of family planning, antenatal care, and HIV care. As access to cervical cancer screening programs improves in resource-poor settings, particularly through the integration of HIV and cervical cancer services, it is important to understand the role of male partner support in women’s utilization of screening and treatment.

**Methods:**

We administered an oral survey to 110 men in Western Kenya about their knowledge and attitudes regarding cervical cancer and cervical cancer screening. Men who had female partners eligible for cervical cancer screening were recruited from government health facilities where screening was offered free of charge.

**Results:**

Specific knowledge about cervical cancer risk factors, prevention, and treatment was low. Only half of the men perceived their partners to be at risk for cervical cancer, and many reported that a positive screen would be emotionally upsetting. Nevertheless, all participants said they would encourage their partners to get screened.

**Conclusions:**

Future interventions should tailor cervical cancer educational opportunities towards men. Further research is needed among both men and couples to better understand barriers to male support for screening and treatment and to determine how to best involve men in cervical cancer prevention efforts.

## Background

There has been a growing interest in integrating men into reproductive health in recent years. An increasing number of studies in developing countries have suggested that involving men in reproductive health improves health outcomes, particularly in regards to family planning, antenatal care, and the prevention of mother-to-child transmission of HIV [1–4]. Men play a role in their partners’ reproductive health experiences in multiple ways, from shared decision-making or granting permission for certain services to providing financial support and transport for health services [4]. In a study in Kenya, women, men, and healthcare providers indicated support for increased male participation in reproductive health, especially in certain areas such as patient education, physician consultation, and financial support [2,4]. However, few studies have examined the influence of male involvement on the utilization of cervical cancer screening.

Despite being preventable with early screening and treatment, cervical cancer is a leading cause of cancer-related mortality among women in developing countries such as Kenya [5]. This high burden of disease is largely a result of lack of access to screening services and inadequate screening uptake due to female patients’ limited knowledge or fears about screening [6–8]. Research has also suggested that a lack of male involvement may be an overlooked obstacle to cervical cancer screening [9,10]. The World Health Organization has recommended integrating men in the prevention of cervical cancer in middle- and low-income countries [11], yet male knowledge and attitudes about cervical cancer screening have not been well-studied in these settings.

Studies of male knowledge about cervical cancer screening in the U.S. indicate that cervical cancer knowledge is low but that men are interested in learning more [12–14]. A study of men in Ghana also demonstrated low awareness about cervical cancer screening, inaccurate knowledge about risk factors and prevention, and stigmatization of cervical cancer diagnosis. Despite this, several men in the study also indicated that they would support screening for their partners but wanted to know more about the disease, screening process, and potential financial costs [15]. In this study, we explored Kenyan men’s knowledge and attitudes about cervical cancer screening.

Family AIDS Care and Education Services (FACES) has been providing cervical cancer screening at an urban HIV clinic in Kenya’s Nyanza Province since 2007 and at several rural Ministry of Health facilities in Nyanza Province since 2013 [16]. During this time, most of the cervical cancer education has been directed towards female clinic attendees. This study measures male clinic attendees’ cervical cancer awareness, knowledge, risk perception, stigma, and willingness to support screening for their female partners. Since many cervical cancer screening programs in Sub-Saharan Africa, including FACES, have targeted HIV positive women through integrated HIV-cervical cancer models and because studies of other integrated programs have suggested a possible association between HIV and cervical cancer stigma, this study also specifically stratified men’s responses by HIV status [17].

## Methods

We conducted this cross-sectional study in April 2013 in both urban and rural areas in Kenya’s Nyanza Province. The urban site was a single clinic providing family-centered HIV care in Kisumu, the capital city of the Nyanza Province. The rural sites were Kenya Ministry of Health clinics that provide general outpatient care as well as family-centered HIV care. Male clinic attendees who had a female partner eligible for cervical cancer screening based on the clinic’s screening guidelines (i.e., non-pregnant women 23–64 years of age) were recruited sequentially with a target of recruiting approximately 100 men, one third from the urban site and two thirds from the rural sites. Those who consented to be in the study were enrolled. A trained interviewer administered an oral survey in English, Kiswahili, or Dholuo, the three official languages spoken in the region. Responses were entered into a tablet computer using Open Data Kit database software [18]. The survey included six sections covering demographic characteristics, general awareness of cervical cancer, specific knowledge about cervical cancer and screening options, perceptions of partner’s risk, stigma, and intention to support screening.

The *Demographic* section of the survey included questions about the participant’s age, education, occupation, relationship status, children, source of health information, history of testing for sexually transmitted infections (STIs) and HIV, and circumcision status. This section also asked about their partner’s use of family planning and prior cervical cancer and breast cancer screening.

The *Awareness* section included five yes/no/“no response” questions asking participants if they had ever heard of cervical cancer, human papillomavirus (HPV), cervical cancer screening, Pap smears, and visual inspection with acetic acid (VIA). The *Knowledge* section consisted of 15 true/false statements that included both facts and common myths about cervical cancer and HPV. A Knowledge Score was then generated, with one point given for each correct answer and no points given for incorrect answers and “I don’t know,” for a maximum possible 15 points.

The *Perception of Risk* section included five yes/no/“I don’t know” questions asking participants whether they think their female partner is at risk for cervical cancer, STIs, HIV, breast cancer, and malaria. The *Stigma* section included two open-ended questions asking how the participants would feel if their partners told them that they had cervical cancer or that they had HIV. This section also asked two yes/no questions about whether participants would consider breaking-up with their partners if they had cervical cancer or had HIV.

Finally, the *Screening Intention* section included three questions asking the men if they would encourage their partners to get screened, who they thought should decide about whether to seek screening (the participant, his partner, or both the participant and partner together), and whether they wanted more information about screening. Two additional open-ended questions then asked what information the participants wanted to learn about cervical cancer and screening and how they could be encouraged to be more involved in their partners’ clinical care.

Bivariate analysis was performed to examine the relationship between demographic characteristics and awareness of cervical cancer screening, Knowledge Scores, perception of partner’s risk, and opinion of who should decide about getting screening. To account for the two main confounders, the results were stratified by HIV status (positive and negative/unknown) and interview site (urban and rural). When conducting bivariate analyses, the appropriate statistical test for association was used after determining the distribution of the data (Pearson’s chi-squared test, Fisher’s exact test, two independent sample t-test, one-way analysis of variance (ANOVA), immediate two-sample test of proportions, and Spearman correlation). This exploratory study was not powered for multivariate analyses.

Open-ended questions were analyzed using a general inductive approach. Responses were reviewed iteratively in order to identify emerging themes and categories. Once a coding scheme was developed, the responses were coded by JMZ. If new codes emerged, the coding scheme was changed and the responses re-reviewed accordingly. The analysis was considered complete when no new themes emerged, indicating that all major themes in the data had been identified. Themes and categories were further refined following discussion among all authors, reducing redundancy and finalizing the most important themes. Themes were quantified and significant statements were identified to represent general themes. Similarities and differences between certain themes and HIV status were also explored, including through use of the immediate two-sample test of proportions.

All data were analyzed using STATA SE statistical software (version 12.1; College Station, Texas). Ethical approval was obtained from the Kenya Medical Research Institute Ethical Review Committee and the University of California, San Francisco Committee on Human Research.

## Results

### Demographic characteristics

One hundred and ten men completed the survey, of which 76 (69.1%) were recruited at rural health facilities and 34 (30.9%) at the urban health center. The median age of participants was 37 years (IQR 28.8–45.7). A majority were married (N = 97, 88.2%) and reported primary school or less as their highest level of education (N = 67, 60.9%). Nearly all (N = 107, 97.3%) men had been tested for HIV at least once. The majority of men (N = 80, 72.7%) were HIV positive, 23 (20.9%) men had tested HIV negative within the last year, and 7 (6.4%) had an unknown HIV status (i.e., never screened or no negative HIV test within the last year). One-fifth of respondents (N = 22, 20.0%) reported that their partner had been previously screened for cervical cancer (Table 1).

**Table 1:** **Demographic characteristics of men with partners eligible for cervical cancer screening in Nyanza Province**, **Kenya** (**n** = **110**)

**Characteristic**	**N (%)**
Age* (years; median [IQR])	37 [28.8–45.7]
***Interview Site***	
Urban	34 (30.9)
Rural	76 (69.1)
***Highest Level of Education Attained***	
Some primary school	67 (60.9)
Some secondary school	34 (30.9)
Beyond secondary school	9 (8.2)
***Occupation***	
Professional/technical/managerial	11 (10.0)
Clerical/sales and services/manual labor	51 (46.4)
Agriculture/fishing	48 (43.6)
***Relationship Status***	
Single	13 (11.8)
Married	97 (88.2)
***Reproductive History***	
# of children fathered* (median [IQR])	3 [1–6]
***Primary source of health information***	
Health facility	58 (52.7)
Radio	50 (45.5)
Church	2 (1.8)
***Prior health seeking behavior***	
Hx of STD testing	57 (51.8)
Hx of HIV testing	107 (97.3)
Hx of male circumcision	47 (42.7)
***HIV Status***	
Positive	80 (72.7)
Negative	23 (20.9)
Unknown**	7 (6.4)
***Reported Partner Family Planning Method***	
Partner does not use FP	24 (21.8)
*Type of family planning used by partner* (*N* = *86*)	
Depo-Provera (injectable)	2 (2.3)
Long-term: IUCD/implant	3 (3.5)
Male Condom	70 (81.4)
Dual (condom + hormonal method)	11 (12.8)
***Partner previously screened for cervical cancer***	22 (20.0)

*IQR*, interquartile range; *IUCD*, intrauterine contraceptive device.

*Variable does not follow a normal distribution.

**“HIV Unknown”: Never screened or no negative test within one year of interview date.

### Awareness and knowledge

Although the majority of men had heard of cervical cancer (N = 101, 91.8%) and cervical cancer screening (N = 98, 89.1%), awareness of specific screening methods and of HPV was low (Table 2). Specific knowledge about cervical cancer was also low, with an average Knowledge Score of 7.2 (SD +/- 3.0). Although 79.1% (N = 87) of men correctly indicated that screening can help prevent cervical cancer, only 51.8% (N = 57) knew that that there is treatment for cervical cancer. Knowledge about risk factors was also low; only 27.3% (N = 30) of men knew that vaginal washing does not decrease cervical cancer risk and only 20.0% (N = 22) knew that cervical cancer is caused by an HPV infection (Table 2).

**Table 2:** **Awareness and knowledge of cervical cancer among men with partners eligible for cervical cancer screening** (**n** = **110**)

	**N (%)**
***Awareness*** (% answered yes)	
Ever heard of cervical cancer	101 (91.8)
Ever heard of cervical cancer screening	98 (89.1)
Ever heard of HPV	48 (43.6)
Ever heard of pap smear	6 (5.5)
Ever heard of VIA	5 (4.6)
Knows someone with cervical cancer	5 (4.6)
***Knowledge*** (% answered correctly)*	
Screening tests look for changes on your cervix that indicate you are at risk for cancer	63 (57.3)
Women should get screened for cervical cancer only if they have symptoms	82 (74.6)
If a woman has abnormal vaginal bleeding, discharge, or pain, she should see a medical provider to get screened for cervical cancer	63 (57.3)
Cervical cancer can be prevented	91 (82.7)
Screening tests can help prevent cervical cancer	87 (79.1)
There is no treatment for cervical cancer	57 (51.8)
***Knowledge of Risk Factors*** (% answered correctly)	
Family planning increases risk	30 (27.3)
HIV increases risk	53 (48.2)
Only HIV + women are at risk	11 (10.0)
Washing inside the vagina decreases risk	30 (27.3)
Screening decreases risk	93 (84.6)
Nothing can prevent cervical cancer because it is fate or the will of God	83 (74.5)
***Knowledge of HPV*** (% answered correctly)	
HPV is an infection that can cause cervical cancer	22 (20.0)
HPV is spread during close contact like during sexual intercourse	24 (21.8)
HPV infection is always symptomatic	7 (6.4)
***Composite Knowledge Score*** (# correct out of 15) (mean +/- SD)	7.2 +/- 3.0

*HPV*, human papillomavirus; *VIA*, visual inspection with acetic acid; *SD*, standard deviation.

*Participants who answered “I don’t know” were counted as incorrect responses.

On bivariate analyses, men with higher education had significantly higher Knowledge Scores (p = 0.003). Men who attained higher than a secondary school education had the highest mean Knowledge Scores (10.2 +/- 2.4), men who completed at least some secondary school had intermediate scores (7.4 +/- 2.6), and men with a primary school education or less had the lowest scores (6.7 +/- 3.0). Knowledge Scores did not differ significantly based on urban/rural location, age, occupation, HIV status, or source of health information.

### Perception of risk

Of the 110 men interviewed, only 48.2% (N = 53) thought their partner was at risk for cervical cancer, while 21.8% (N = 24) did not think their partner was at risk for cervical cancer, and 30.0% (N = 33) were not sure. This finding was similar to the perceived risk of a sexually transmitted infection (51.8%, N = 57) but significantly lower (p < 0.001) than perceived risk for HIV (N = 96, 87.3%), malaria (N = 95, 86.4%), and breast cancer (N = 91, 82.7%). (Table 3) Among the 30 men who had a negative or unknown HIV status, 66.7% (N = 20) thought their partner was at risk for HIV. Perception of cervical cancer risk did not differ significantly by HIV status or urban/rural location.

**Table 3:** **Perceptions of partner disease risk among men with female partners eligible for cervical cancer screening** (**n** = **110**)

***I think my partner is at risk for:***	**Yes N (%)**	**No N (%)**	**I don**’**t know N (%)**
Cervical cancer	53 (48.2%)	24 (21.8%)	33 (30.0%)
Sexually transmitted disease (i.e. gonorrhea, chlamydia)	57 (51.8%)	42 (38.2%)	11 (10.0%)
Breast cancer	91 (82.7%)	2 (1.8%)	17 (15.5%)
HIV	96 (87.3%)	11 (10.0%)	3 (2.7%)
Malaria	95 (86.4%)	14 (12.7%)	1 (0.9%)

### Stigma

When asked how they would feel about a partner’s diagnosis of cervical cancer, respondents tended to describe emotional states, the most common being “sad” (26.4% of responses) and “pain” (12.7% of responses). Negative emotions were also widely cited, including “bad”, “disappointed”, “stressed”, and “shocked” (20.9% of responses). Regardless of their emotional reactions, 21.8% of men said they would try to assist their partners in the event of a cervical cancer diagnosis, such as by seeking help from a health facility or searching for treatment. One respondent said, “Try my best to take her to the hospital”, and another said, “I can look for ways to help her”. Sixteen (14.5%) respondents said that a partner’s cervical cancer diagnosis would be “normal” or that they would “accept” the diagnosis. Three (2.7%) men expressed concern that treatment would be expensive, and two (1.8%) men reported that they would consider breaking-up with their partner if the partner was diagnosed with cervical cancer.

When asked how they would feel if their partner was diagnosed with HIV, some men also described emotional states, including “pain” (7.3% of responses), “sad” (4.5% of responses), and negative emotions such as “bad”, “stressed”, “disappointed”, and “afraid” (10.9% of responses) (Table 4). Notably, fewer men reported negative emotional states in response to HIV compared to cervical cancer. Acceptance and perceived normalcy of a partner’s HIV diagnosis was higher than for cervical cancer. Of the 80 HIV positive men, 66 (82.5%) reported that this would be “normal”, as well as eight (26.7%) of the 30 HIV status negative/unknown men. Five men (4.5%) said they would consider ending the relationship if their partner was diagnosed with HIV, and two men (1.8%) were unsure. An HIV-positive status significantly predicted acceptance of a partner’s HIV diagnosis (p = 0.009), though HIV status was not a significant predictor of acceptance of a partner’s cervical cancer diagnosis (p = 0.061).

**Table 4:** **Attitudes towards cervical cancer among men with partners eligible for cervical cancer screening** (**n** = **110**)*

***How would you feel if your partner told you she had cervical cancer?***	
Theme	Frequency (% of responses)
***Emotion***	
Sad	29 (26.4)
Pain	14 (12.7)
Bad (includes bad, disappointed, stressed, shocked, afraid, depressed, dismay)	23 (20.9)
Other (includes sympathy, strange, amused)	9 (8.2)
***Outlook***	
Acceptance of diagnosis	5 (4.5)
Diagnosis is normal	11 (10.0)
***Behavior***	
Seek help for partner	24 (21.8)
***Cervical Cancer Beliefs***	
Cervical cancer is treatable	3 (2.7)
No cure for cervical cancer	1 (0.9)
Cervical cancer expensive to treat	3 (2.7)
***How would you feel if your partner told you she had HIV?***	
Theme	Frequency (% of responses)
***Emotion***	
Pain	8 (7.3)
Sad	5 (4.5)
Sorry (includes sorry, remorse)	4 (3.6)
Bad (includes bad, stressed, disappointed, afraid, angry, depressed)	12 (10.9)
Other (includes astonished, calm, strange)	5 (4.5)
***Outlook***	
Acceptance of diagnosis	15 (13.6)
Diagnosis is normal	59 (53.6)
***Behavior***	
Seek help for partner	5 (4.5)
Talk w/partner	1 (0.9)
Get tested	3 (2.7)

*Answer responses (n = 110) were qualitatively analyzed using a general inductive approach, and frequencies were computed based on how often the themes occurred in the responses. Answer responses could include more than one theme.

### Screening intention

All of the men surveyed (N = 110, 100%) reported that they would allow and even encourage their partner to get screened for cervical cancer. Forty-one men (37.3%) felt that the decision to get screened should be made by their partner alone, while 68 (61.8%) felt that the decision should be made jointly; only one man reported that he should make the decision alone. In bivariate analyses, men from rural areas were significantly more likely to think that screening should be a joint decision compared to men from the urban center (p = 0.017).

All of the men surveyed (N = 110, 100%) said they wanted more information about cervical cancer screening. The most common information sought regarded causes of cervical cancer (68.2% of responses), followed by treatment (35.5%) and symptoms (30.0%). Nearly one-fifth of respondents were concerned about whether they (and men generally) could be exposed to or at risk of cervical cancer (18.2% of responses). Questions about transmission came up in 20% of responses, though it was unclear whether respondents were concerned with their partners’ risk of transmission or their own. Nearly all respondents (N = 104, 94.5%) reported that increasing their knowledge would help make them more involved in their partner’s clinical care.

## Discussion

Men’s knowledge and attitudes about cervical cancer screening have been understudied, particularly in sub-Saharan Africa. To our knowledge, this is the first exploratory analysis of men’s knowledge about cervical cancer in East Africa, sampling men who attended both rural and urban clinics. We found specific knowledge about cervical cancer risk factors, prevention, and treatment to be low among the men in our study, which is similar to findings from a study of male focus groups in Ghana [15]. Men with higher levels of education demonstrated significantly greater knowledge about cervical cancer. This study used the same knowledge scale administered to women in the same health facilities around the same time period; we found that the average men’s Knowledge Score (7.2 +/- 3.0) was similar to but slightly lower than women’s average score (8.6 +/- 2.4) [19].

Despite their low cervical cancer knowledge, all men expressed an interest in learning more and many asked specific questions about cervical cancer and how it relates to them. All men also said that they would encourage their partner to get screened and many wanted to be involved in the decision-making process with their partner. This interest in reproductive health education and willingness to get more involved in their partner’s healthcare is similar to what has been reported for male involvement in family planning and antenatal care in this region [4]. It is important to recognize, however, that while men may want to support their partners, they may face challenges in getting time off to attend clinic visits, hostility from healthcare staff, or difficulty providing financial support to pay for transportation or clinical services, as suggested in other studies [2,4]. In our study’s open-ended survey responses, some participants expressed fears about how they would be able to support their partners after a diagnosis of cervical cancer.

Notably, knowledge and acceptance of HIV appeared higher among study participants compared to cervical cancer. While this study was not designed to evaluate such a difference, we speculate that it reflects the high percentage of HIV-positive men in our sample, our recruitment from clinics providing HIV care, the high prevalence of HIV in the region, and the success of HIV-related education and outreach campaigns in the community. Higher HIV knowledge and acceptance may thus reflect the positive impact of health messaging and improved health infrastructure that could serve as a model for cervical cancer prevention.

This study is limited by social desirability bias, perhaps reflected in the fact that all men responded that they would encourage their partners to get screened. Given the homogeneity of the responses, we were unable to examine potential predictors of male support. Additionally, the representativeness of our sample is limited given the high percentage of HIV-positive males and the fact that all men sampled were clinic attendees. Our participants may have been more educated about reproductive health issues and more willing to support their partners in getting screened than men in the general community.

## Conclusions

Given men’s low cervical cancer knowledge and strong interest in learning more, future interventions should increase cervical cancer education opportunities that are tailored towards men. Studies are also needed to better understand other barriers to male partners’ support of cervical cancer screening and support for a partner after a diagnosis is made, particularly regarding issues such as financial support, transportation, getting time off work, limited space in exam rooms, and provider attitudes towards male involvement. Finally, couples-level data combining screening records and the knowledge and attitudes of both parties are needed in order to determine how best to involve men to improve partner screening and follow-up rates.
